# Effectiveness and Cost-Effectiveness of Sequential Treatment of Patients with Chronic Myeloid Leukemia in the United States: A Decision Analysis

**DOI:** 10.1155/2015/982395

**Published:** 2015-12-10

**Authors:** Ursula Rochau, Martina Kluibenschaedl, David Stenehjem, Kuo Kuan-Ling, Jerald Radich, Gary Oderda, Diana Brixner, Uwe Siebert

**Affiliations:** ^1^Institute of Public Health, Medical Decision Making and Health Technology Assessment, Department of Public Health, Health Services Research and Health Technology Assessment, UMIT-University for Health Sciences, Medical Informatics and Technology, Eduard Wallnoefer Center 1, 6060 Hall in Tirol, Austria; ^2^Area Health Technology Assessment, ONCOTYROL-Center for Personalized Cancer Medicine, Karl-Kapferer-Straße 5, 6020 Innsbruck, Austria; ^3^Department of Pharmacotherapy, University of Utah, 30 South 2000, Salt Lake City, UT 84112, USA; ^4^Huntsman Cancer Institute, University of Utah Hospitals & Clinics, 2000 Circle of Hope, Salt Lake City, UT 84112, USA; ^5^Clinical Research Division, Fred Hutchinson Cancer Research Center, 1100 Fairview Avenue N, Seattle, WA 98104, USA; ^6^Program in Personalized Health, University of Utah, 15 North 2030 East, Room 2110, Salt Lake City, UT 84112, USA; ^7^Center for Health Decision Science, Department of Health Policy and Management, Harvard T.H. Chan School of Public Health, 718 Huntington Avenue, Boston, MA 02215, USA; ^8^Institute for Technology Assessment, Department of Radiology, Massachusetts General Hospital, Harvard Medical School, 101 Merrimac Street, Boston, MA 02114, USA

## Abstract

Currently several tyrosine kinase inhibitors (TKIs) are approved for treatment of chronic myeloid leukemia (CML). Our goal was to identify the optimal sequential treatment strategy in terms of effectiveness and cost-effectiveness for CML patients within the US health care context. We evaluated 18 treatment strategies regarding survival, quality-adjusted survival, and costs. For model parameters, the literature data, expert surveys, registry data, and economic databases were used. Evaluated strategies included imatinib, dasatinib, nilotinib, bosutinib, ponatinib, stem-cell transplantation (SCT), and chemotherapy. We developed a Markov state-transition model, which was analyzed as a cohort simulation over a lifelong time horizon with a third-party payer perspective and discount rate of 3%. Remaining life expectancies ranged from 5.4 years (3.9 quality-adjusted life years (QALYs)) for chemotherapy treatment without TKI to 14.4 years (11.1 QALYs) for nilotinib→dasatinib→chemotherapy/SCT. In the economic evaluation, imatinib→chemotherapy/SCT resulted in an incremental cost-utility ratio (ICUR) of $171,700/QALY compared to chemotherapy without TKI. Imatinib→nilotinib→chemotherapy/SCT yielded an ICUR of $253,500/QALY compared to imatinib→chemotherapy/SCT. Nilotinib→dasatinib→chemotherapy/SCT yielded an ICUR of $445,100/QALY compared to imatinib→nilotinib→chemotherapy/SCT. All remaining strategies were excluded due to dominance of the clinically superior strategies. Based on our analysis and current treatment guidelines, imatinib→nilotinib→chemotherapy/SCT and nilotinib→dasatinib→chemotherapy/SCT can be considered cost-effective for patients with CML, depending on willingness-to-pay.

## 1. Introduction

Chronic myeloid leukemia (CML) is the third most common type of leukemia [1]. In 2014, it is estimated that 5,980 people will be diagnosed and 810 people will die from CML in the United States (US) [2]. The disease course consists of three phases [3]. Although there are some differences in the clinical and pathological definitions of these phases, the progression from chronic phase (CP) to accelerated phase (AP) and AP to blast phase (BP) is a decisive factor for prognosis and treatment [4]. All therapies for CML (interferon, tyrosine kinase inhibitors (TKIs), or allogeneic stem-cell transplantation (SCT)) are far superior for CP than AP/BC. The approval of TKIs enabled considerable changes in the treatment of newly diagnosed CML patients [5] by turning this type of blood cancer into a chronic disease with long periods of remission [6]. However, the only known curative treatment option still remains a SCT [3].

The price of imatinib has tripled since 2001 in the US, from $30,000 per year to $92,000 [7]. Patients in the US often pay out-of-pocket approximately 20% of the drug price, resulting in 10% of patients who are unable to afford prescription drugs [8]. Besides imatinib, there are five other drugs approved by the FDA for CML: nilotinib, dasatinib, bosutinib, ponatinib, and omacetaxine. Each of these therapies costs approximately $118,000–$138,000 annually [7]. Currently imatinib, nilotinib, and dasatinib are FDA approved for the first-line treatment of CP-CML and evidence exists for the short-term superiority of dasatinib and nilotinib compared to imatinib, based on 12-month rates of complete cytogenetic remission, major molecular response, and progression to advanced phase disease. However, some cases of CP-CML become intolerant or resistant to TKI therapy, and in these cases, switching to a second-line agent is necessary. However the optimal sequence of these agents balancing effectiveness and cost has not been demonstrated.

When faced with choosing between the number of available treatment strategies with the goal to balance benefits, harms, and costs, decision-analytic modeling can be used as a supportive instrument in the decision making process [9, 10]. Its techniques allow us “to compare the expected consequences of different strategies after considering all relevant events and complications with their probabilities and weighing all relevant clinical outcomes and costs” [9].

Hence, we developed and updated a Markov state-transition model [11], to identify which sequential treatment strategy provides the optimal balance between clinical effectiveness and cost-effectiveness for CP-CML within the US health care context.

## 2. Methods

### 2.1. Model Design

To represent available treatment options within the US health care context, 18 different sequential treatment strategies (Table 1) consisting of combinations of first-generation, second-generation, and third-generation TKIs were considered. With the exception of first-line bosutinib, all included TKIs are currently approved by the US Food and Drug Administration [12], as either second-line treatment option or both second-line and first-line. As there is currently a trial for first-line bosutinib ongoing [13] and bosutinib might be approved as first-line therapy for CP-CML patients in the future, we included treatment strategies with first-line bosutinib. To compare the effect of each strategy with “no treatment,” a baseline strategy that includes chemotherapy only was incorporated into the model.

**Figure 1:**
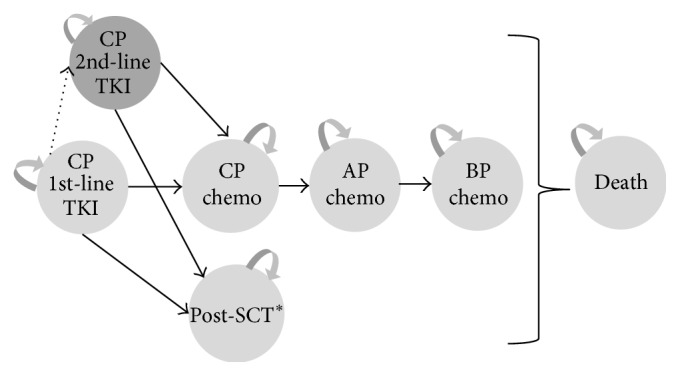
State-transition diagram of the Markov state-transition model. AP, accelerated phase; BP, blast phase; CP, chronic phase; SCT, stem-cell transplantation; and TKI, tyrosine kinase inhibitor. ^*∗*^split in two states with different quality of life, costs, and survival depending on graft versus host and complications after SCT.

We expanded, updated, and adapted our previously developed Markov state-transition model [11, 48] to the clinical setting in the US. We followed international guidelines to develop, analyze, and report the model and results [49–53]. In the model, all patients begin with a first-line TKI treatment in CP. Subsequently, patients can either remain in the same health state or move after first-line TKI failure to second-line TKI treatment in CP, chemotherapy, or receive a SCT. As the disease progresses, patients can move to AP and BP. While patients can die in each health state from other reasons, dying from CML is just possible in BP [11]. The state-transition diagram in Figure 1 shows the different health states included in the Markov model. Patients cycle through the nine mutually exclusive and collectively exhaustive health states in monthly cycles over a lifelong time horizon.

The Markov model was analyzed as a cohort simulation. For model evaluation, the absolute and incremental outcome measures life years (LYs), quality-adjusted life years (QALYs), and costs, as well as incremental cost-effectiveness ratios (ICERs in $/LY gained) and incremental cost-utility ratios (ICURs in $/QALY gained) were used. A third-party payer perspective was adopted and a discount rate of 3% was applied for both costs and health outcomes [54].

The following important assumptions and simplifications were made: in advanced phases of the disease, chemotherapy was the only treatment option. Additionally, the sequential application of only two different TKIs was considered in the model and dose modification was not allowed within the model. After receiving SCT, patients were assumed to either survive without relapse or die.

The model was programmed and analyzed in TreeAge 2015 (TreeAge Software, Inc. Williamstown, MA, USA).

### 2.2. Model Input Parameter

#### 2.2.1. Natural History Model Parameters

In the US, the median age at diagnosis for CML is approximately 64 years [55]; thus, the model cohort starting age was determined to be 64 years. The proportion of male (62%) and female CML patients was derived from the Huntsman Cancer Institute Tumor Registry [56]. For non-CML mortality, data from US life tables [57] were used.

#### 2.2.2. Transition Probabilities

Data for natural history, effectiveness, adverse events, and costs are reported in Table 2. For the estimation of the duration of first-line treatment, Weibull functions were fitted to the respective trial data (see Table 2). To estimate the probability of continuing second-line TKI therapy, we fitted exponential curves due to the lack of long-term data (see Table 2). Within the framework of US expert survey, we ascertained the proportion of patients receiving transplantation after TKI failure dependent on patients age [58]. The proportion of patients receiving a SCT from a related donor (68%) compared to nonrelated donors was derived from the Huntsman Cancer Institute Tumor Registry and the University of Utah Enterprise Data Warehouse [56].

#### 2.2.3. Utilities

Besides survival, we considered preference-based health outcomes. Survival was adjusted for health-related quality of life (QoL) using quality of life indices (i.e., utilities). Life years were multiplied by utilities to derive QALYs. Utility values can range from one (perfect health) to zero (death) [59]. Utility values for patients in the CP, AP, and BP were elicited from patients in the IRIS study using the EQ-5D [40, 41, 60]. For patients who received a SCT, data were used from a study that interviewed clinical experts using the standard gamble technique to value the health states of patients after SCT [43]. Age-dependent utility values of the general population in the US were derived from a survey conducted by Fryback et al. 1993 [42]. We compared general population utilities with utilities from the patients in the IRIS trial and derived relative disutilities for each health state (u(CML patients)/u(general population)). The utilities in the general population in each age group were then weighted with the relative disutility for each CML patient according to each health state. For details see Rochau et al. 2014 [11].

#### 2.2.4. Costs

The costing index year for the analysis was 2014. All cost data were adjusted for inflation to US dollars for the price level of June 2014 using the consumer price index (CPI) for medical services and the CPI for medical care commodities [61, 62].

Drug prices were derived from the Redbook [44]. Dosages are in line with those doses given in clinical trials (Table 2). The monthly therapy costs for each phase were derived from two publications of Reed and colleagues [40, 45]. Reed et al. used DRG data and Medicare nonfacility CPT payment. Resource utilization data in Reed et al. are based on data collected from the International Randomized Interferon versus STI571 (IRIS) Study. For allogeneic SCT and for treatments of adverse events, DRGs derived with HCUPnet [47] were used. Based on the advice of clinical experts, we assumed that all grade 3 and 4 adverse events would be treated in the hospital. Adverse events on first- and second-line TKI treatment were derived from the respective trials where the effectiveness data were derived from (see Table 2). Additionally, for simplification, only adverse events that occurred with a frequency of at least 5% and only grades 3 and 4 were considered. Table 2 gives an overview on the model parameters.

### 2.3. Base-Case and Sensitivity Analyses

#### 2.3.1. Base-Case Analysis

Comparative clinical effectiveness was estimated as survival and quality-adjusted survival for each treatment strategy. In the cost-effectiveness analysis, we compared clinical outcomes to costs and calculated incremental cost-effectiveness ratios and incremental cost-utility comparing the different strategies. To calculate incremental ratios, first, strategies were ordered according to their costs (i.e., starting with the least expensive strategy). Subsequently, strategies that were less effective and more or equally expensive as the comparator were excluded due to dominance. After removal of the dominated strategies, ICERs and ICURs were calculated by applying the following formula: [Costs(Strategy1) – Costs(Strategy2)]/[Effectiveness(Strategy1) – Effectiveness(Strategy2)]. Afterwards, the ICERs and ICURs were compared and strategies that were weakly dominated were excluded as well. Weak dominance or “extended dominance rules out any strategy with a higher incremental cost-effectiveness ratio (ICER), which is greater than that of a more effective strategy. That is, extended dominance applies to strategies that are not cost-effective because another available strategy provides more units of benefit at a lower cost per unit of benefit [63].”

#### 2.3.2. Sensitivity and Scenario Analysis

In 2015, it is expected that imatinib will lose patent protection [7]; therefore, we analyzed a price reduction of imatinib between 40% and 60% [64]. Since the proportion of patients receiving a SCT depending on age was estimated by only a few experts and can differ largely by clinician and practice setting, we tested in scenario analysis the model without the possibility of SCT to analyze if the decision would be different. As the duration of second-line treatment was fitted and extrapolated from rather few data points, we evaluated the impact of varying the duration of second-line treatment on the decision.

## 3. Results

### 3.1. Base-Case Analysis

In the comparative effectiveness analysis, remaining life expectancies (undiscounted) ranged from 5.4 years (3.9 QALYs) to 14.4 years (11.1 QALYs). Our analyses showed a large gain in life expectancy when using a treatment strategy that includes a TKI instead of only chemotherapy. The least effective strategy including a TKI was bosutinib→chemotherapy/SCT. Compared to chemotherapy alone, patients were expected to gain on average 4.7 QALYs (3.9 QALYs versus 8.6 QALYs) or 5.9 LYs (5.4 LYs versus 11.3 LYs). All remaining strategies range only from 8.6 QALYs to 11.1 QALYs (11.3 LYs to 14.4 LYs). The most effective treatment strategy without second-line TKI was nilotinib→chemotherapy/SCT with an expected remaining life expectancy of 12.8 years (9.7 QALYs). The most effective strategy was nilotinib→dasatinib→chemotherapy/SCT with 14.4 LYs (11.1 QALYs) (Table 1 Supplementary Appendix in Supplementary Material available online at http://dx.doi.org/10.1155/2015/982395).

**Figure 2:**
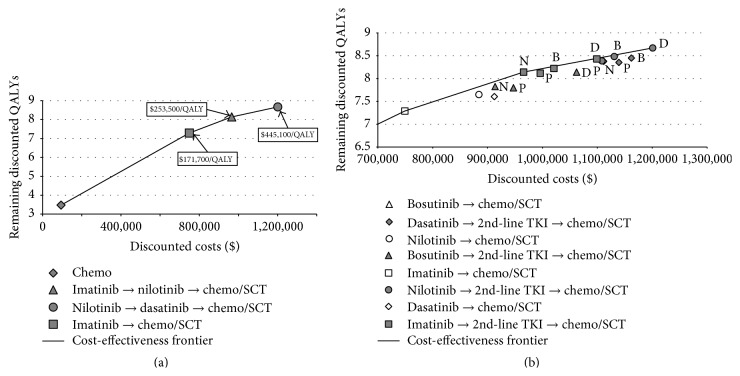
Cost-effectiveness plane base-case analysis. (a) Cost-effectiveness frontier including only nondominated strategies. (b) Cost-effectiveness frontier including all strategies except chemotherapy alone. Chemo: chemotherapy; QALYs: quality-adjusted life years; SCT: stem-cell transplantation. Letters next to the symbols in (b) indicate the second-line TKI: B: bosutinib; D: dasatinib; P: ponatinib; N: nilotinib; the shape of the symbols explained in the legend beneath the graph indicates the first-line TKI. The cost-effectiveness plane presents simultaneously costs (*x*-axis) and health outcomes (*y*-axis). Strategies on the left lower corner of the cost-effectiveness plane are less expensive and less effective compared to strategies on the right upper corner. After eliminating dominated and weekly dominated strategies, four strategies (see (a)) remained defining the so-called cost-effectiveness frontier (i.e., the line in Figure 2): (1) chemotherapy, (2) imatinib→chemotherapy/SCT, (3) imatinib→nilotinib→chemotherapy/SCT, and (4) nilotinib→dasatinib→chemotherapy/SCT.

The cost-effectiveness plane in Figure 2 shows the results of considering costs as well as health outcomes. The costs are shown on the *x*-axis and the health outcomes measured as QALYs on the *y*-axis. Strategies on the left lower corner of the cost-effectiveness plane are less expensive and less effective compared to strategies on the right upper corner. After eliminating dominated and weakly dominated strategies, four strategies remained defining the so-called cost-effectiveness frontier (i.e., the line in Figure 2): (1) chemotherapy, (2) imatinib→chemotherapy/SCT, (3) imatinib→nilotinib→chemotherapy/SCT, and (4) nilotinib→dasatinib→chemotherapy/SCT. Dominated strategies can be easily identified in Figure 2 as they lie below the cost-effectiveness frontier (e.g., bosutinib→dasatinib→chemotherapy/SCT).

The remaining nondominated strategies resulted in the following ICERs and ICURs. Imatinib without second-line TKI came to an ICUR of $171,700 per QALY gained (ICER $137,900 per LY gained) compared to the baseline strategy chemotherapy. Imatinib→nilotinib→chemotherapy/SCT yielded an ICUR of $253,500/QALY (ICER of $260,800/LY) compared to imatinib→chemotherapy/SCT. Nilotinib→dasatinib→chemotherapy/SCT had an ICUR of $445,100/QALY (ICER of $299,800/LY) compared to imatinib→nilotinib→chemotherapy/SCT (Table 3).

### 3.2. Sensitivity and Scenario Analyses

We investigated the scenario of generic drug pricing of imatinib.  Table 4 shows the results of a price decrease of imatinib to 60% (B) of the original drug price as well as a price reduction to 40% (C) of the original drug price. Based on these price reductions, strategies including imatinib became more cost-effective and resulted in lower ICURs (e.g., ICUR of the strategy imatinib→chemotherapy/SCT was in the base-case analysis $171,700/QALY and dropped to $109,000/QALY and $77,600/QALY in the scenarios of 60% and 40% of imatinib's original price). Additionally, the strategy of imatinib→dasatinib→chemotherapy/SCT was no longer dominated.

We performed best- and worst-case scenario assuming first that all second-line treatments have the same effectiveness as dasatinib (most effective second-line treatment in our analysis) and second a worst-case analysis assuming all second-line treatments have the same effectiveness as second-line ponatinib, which has demonstrated the least second-line effectiveness on average in our analysis (see Table 2 Supplementary Appendix). These worst- and best-case scenarios showed that the results are robust. The nondominated strategies and their rankings did not change. The deviation of the ICURs from the base-case ICURs varied between 2% and 17% (Table 2 Supplementary Appendix).

In another scenario analysis, we evaluated the impact of excluding SCT as an option. Excluding SCT as a treatment option does not have a huge impact on our outcome. Only one additional strategy becomes nondominated (imatinib→dasatinib→chemotherapy/SCT), and the ICURs for imatinib→chemotherapy/SCT and imatinib→nilotinib chemotherapy/SCT change slightly.

## 4. Discussion

Several TKIs are approved and recommended in guidelines for first- and further-line treatment of CML in the US. Our study is the first one that analyzed 18 different combination strategies over a lifelong time horizon. When comparing the health outcomes, we showed that adding a TKI rather than only using chemotherapy increased the life expectancy substantially. Additionally, sequential treatment, as recommended by current treatment guidelines, brings another additional gain in life expectancy. When considering costs as well, two nondominated strategies including a second-line TKI remained on the cost-effectiveness frontier: imatinib→nilotinib→chemotherapy/SCT and nilotinib→dasatinib→chemotherapy/SCT. However, if imatinib loses patent protection, anticipated in 2015 [7], the price is expected to drop significantly. The scenario analyses we conducted on generic pricing, with imatinib priced at 40%–60% of current costs, show that imatinib→nilotinib→chemotherapy/SCT remains an attractive nondominated strategy, whereas the strategy nilotinib→dasatinib→chemo/SCT results in rather high ICERs. Starting with imatinib followed by a second-line TKI, such as nilotinib, is also a treatment strategy supported by the current NCCN guidelines [65]. However, even higher price drops up to 90% decline of imatinib's original price might be possible [66] and consecutive price changes in the second-generation TKIs need to be taken into account to derive further recommendations [66].

In the US, there is no commonly accepted willingness-to-pay threshold as, for example, in the UK (20,000–30,000 £/QALY) [67] or the Netherlands (80,000 €/QALY) [68]. Occasionally, a WTP threshold of $50,000/QALY [69] is applied or by definition of the WHO three times the gross domestic product per disability-adjusted life year [70]. The $50,000/QALY threshold was introduced in 1982 [71] and after updating it to 2014, it would be $122,755/QALY [72] today. Braithwaite et al. suggested a threshold range of $183,000/LY to $264,000/LY saved [73] (2014: $235,630/LY to $339,926/LY). Our strategy imatinib→nilotinib→chemotherapy/SCT (ICER of $260,800/LY) would lie close to the lower threshold range. The remaining nondominated strategy including a second-line TKI, nilotinib→dasatinib→chemo/SCT ($299,800/LY), would fall within the threshold range. The ACC/AHA (American College of Cardiology/American Heart Association) Task Forces suggested recently that if “the cost per QALYs gained was <$50,000,” the therapy would be given a high level of value recommendation and if “the cost per QALYs gained was >$150,000, it would be given a low level of value recommendation [74]. The relatively high ICERs compared to willingness-to-pay thresholds of other countries are consistent with the increasing prices for cancer drugs in general as well as for CML specifically [7, 75]. “Of the 12 drugs approved by the FDA for various cancer indications in 2012, 11 were priced above $100 000 per year [7].” For example, in the UK, a special cancer drug fund was initiated to pay for cancer drugs that are not routinely available within the national health system [76].

Further modeling studies resulted in similar recommendations. Our Austrian analysis [48] resulted in a comparable ranking of the strategies as the application to the US context. However, the Austrian analysis did not include the newly approved ponatinib and bosutinib. The recommended strategy derived from the Austrian analysis was imatinib→nilotinib→chemotherapy/SCT with an ICUR of 131,100 €/QALY (corresponds to 163,188 $/QALY in 2014). Differences are explained by higher drug prices in the US, higher costs for SCT, and probabilities specific to the national context, such as the probability of receiving a SCT after TKI failure dependent on age.

Additionally, we compared our model results to models described in a recently published review on CML [11]. None of the models identified in the review included ponatinib or bosutinib; and only three included nilotinib or dasatinib [1, 3, 77–79]. Ghatnekar et al. [1] as well as Hoyle et al. [77–79] compared treatment strategies for CML patients in CP that were resistant to imatinib; they were also not comparable to our analysis. Pavey and colleagues [3] evaluated the cost-effectiveness of dasatinib, nilotinib, and imatinib for the first-line treatment of CML followed by second-line nilotinib [3]. They compared several scenarios under various assumptions. In accordance with our analyses, they found that “first-line dasatinib is predicted to provide very poor value for money compared with first-line imatinib regardless of the model structure [3]”.

A significant strength of our analysis is the systematic, evidence-based, and comprehensive evaluation of 18 different treatment strategies and the inclusion of newly approved CML treatments, such as ponatinib and bosutinib. It would be hardly possible to compare 18 different treatment strategies in a randomized controlled clinical trial with sufficient sample size. Furthermore, we extrapolated short-term trial data to a lifelong time horizon and adjusted the survival for QoL to generate comprehensive patient-relevant outcomes. Evaluating and reporting the generic measure “quality-adjusted life years” help to optimize the benefit-harm tradeoff as it combines the treatment strategies' short- and long-term effects on duration and quality of life. Furthermore, evaluation of QALYs helps to direct health care resources most efficiently as comparisons across disease are possibly opposed to reports on disease specific outcomes measures, such as incidence of complications or response rates [80]. In particular, when only evaluating survival, “any impact on the quality of life associated with an intervention is ignored” [80]. On the other hand, often comprehensive and QoL data are not available, the results may differ when different stakeholders are asked (patients versus public versus physicians), and preferences may also be dependent on the setting and vary immensely between different individuals or societies. Therefore, we presented both survival and quality-adjusted survival enabling health policy decision makers and clinicians to compare the different treatment strategies within the disease on different levels but also across diseases and to national willingness-to-pay threshold values.

Our study has several limitations. First, there were no utilities available specifically for the US setting and also no utilities specific to each treatment line. When comparing QALYs, this is a major limitation that can only be solved by conducting utility studies. However, we do also report the results for LYs without adjustment for QoL and these results support the analyses including QALYs. Second, we did not include third-line TKI treatment. This might have influenced the absolute number of LYs but should not have a big influence in comparing the different combination strategies as all of them did not include third-line TKI treatment. Another assumption was that patients cannot relapse after SCT. We considered a higher mortality compared to the general population but not a relapse after SCT. This might have led to a slightly better outcome across all strategies; however, it should not have biased the results between the different strategies. The effectiveness data were derived from pivotal clinical trials that might not represent the real world target population. However, as the effectiveness data was applied consistently across the treatment strategies it would be unlikely to influence the differences observed. Treatment patterns and access to treatment might depend on various factors, such as physician preferences, hospital policies, or insurance coverage. Therefore, we analyzed comprehensively 18 different strategies. Additionally, the model is flexible to be adapted to other settings in the future. Another limitation of our modeling approach is that the choice of the first-line treatment might also be influenced through the long-term safety results of imatinib and recent concerns that, for example, the newer TKIs might lead to severe complications, such as pleural effusion, arterial hypertension, or vascular events [81, 82]. Furthermore, we did not consider specific mutations and more personalized aspects for treatment decisions; this will be incorporated in further developments of the model as well as the option of stopping TKI. Preliminary results of trials testing the stopping of TKI treatment are showing promise [83, 84].

## 5. Conclusion

In conclusion, the model results suggest that imatinib followed by second-line nilotinib and nilotinib followed by second-line dasatinib are candidates for cost-effective sequential treatment strategies among those including a second-line TKI for chronic phase CML in the US. The decision on the cost-effectiveness has to be made in the context of individual or society's willingness-to-pay. These results may be used to support CML treatment decision making by clinicians and patients.

## Supplementary Material

The supplementary appendix gives an additional overview on modeling results. Table S1 shows the results of the effectiveness results of the base-case analysis without discounting and Table S2 provides the results of a scenario analysis varying the second-line TKI effectiveness.

## Figures and Tables

**Table 1:** Sequential treatment strategies.

First-line TKI	Second-line TKI	Chemotherapy/stem-cell transplantation
Chemotherapy^*∗*^	—	—
Imatinib	—	Chemotherapy/stem-cell transplantation
Bosutinib	—	Chemotherapy/stem-cell transplantation
Dasatinib	—	Chemotherapy/stem-cell transplantation
Nilotinib	—	Chemotherapy/stem-cell transplantation
Imatinib	Bosutinib	Chemotherapy/stem-cell transplantation
Imatinib	Dasatinib	Chemotherapy/stem-cell transplantation
Imatinib	Nilotinib	Chemotherapy/stem-cell transplantation
Imatinib	Ponatinib	Chemotherapy/stem-cell transplantation
Bosutinib	Dasatinib	Chemotherapy/stem-cell transplantation
Bosutinib	Nilotinib	Chemotherapy/stem-cell transplantation
Bosutinib	Ponatinib	Chemotherapy/stem-cell transplantation
Dasatinib	Bosutinib	Chemotherapy/stem-cell transplantation
Dasatinib	Nilotinib	Chemotherapy/stem-cell transplantation
Dasatinib	Ponatinib	Chemotherapy/stem-cell transplantation
Nilotinib	Bosutinib	Chemotherapy/stem-cell transplantation
Nilotinib	Dasatinib	Chemotherapy/stem-cell transplantation
Nilotinib	Ponatinib	Chemotherapy/stem-cell transplantation

TKI: tyrosine kinase inhibitor.

^*∗*^Hydroxyurea.

**Table 2:** Data for natural history, effectiveness, adverse events, and costs.

Input parameter	Value	Source (references)
*Treatment effectiveness*		
Probability of staying on 1st-line chemotherapy	Weibull, shape: 1.17, scale: 53.15	[14, 15]
Probability of staying on 1st-line bosutinib	Weibull, shape: 0.92, scale: 54.79	[13, 16–18]
Probability of staying on 1st-line dasatinib	Weibull, shape: 0.92, scale: 102.4	[19–21]
Probability of staying on 1st-line imatinib	Weibull, shape: 0.92, scale: 79.65	[13, 16–24]
Probability of staying on 1st-line nilotinib	Weibull, shape: 0.92, scale: 106	[22–24]
Probability of staying on 2nd-line bosutinib	Exponential, 0.97	[25, 26]
Probability of staying on 2nd-line dasatinib	Exponential, 0.98	[27–29]
Probability of staying on 2nd-line nilotinib	Exponential, 0.97	[30–33]
Probability of staying on 2nd-line ponatinib	Exponential, 0.97	[34–37]
Probability of staying in CP on chemotherapy after TKI failure	Exponential, 0.01	[38]
Probability of staying in AP on chemotherapy	Exponential, 0.11	[39]
Probability of dying from CML in BP on chemotherapy	Exponential, 0.09	[38]
*Utilities*		
Chronic phase	0.92 × (age-dependent utility general population)	[40–42]
Accelerated phase	0.79 × (age-dependent utility general population)	[41, 42]
Blast phase	0.57 × (age-dependent utility general population)	[41, 42]
After SCT without GvHD	0.98 × (age-dependent utility general population)	[42, 43]
After SCT with GvHD	0.9 × (age-dependent utility general population)	[42, 43]
*Drug cost*		
Imatinib 1st line 400 mg qd	$10,057.04 per month	[44]
Dasatinib 1st line 100 mg qd	$11,021.20 per month	[44]
Nilotinib 1st line 300 mg bid	$10,436.08 per month	[44]
Bosutinib 1st line 500 mg qd	$11,277.36 per month	[44]
Dasatinib 2nd line 100 mg qd	$11,021.20 per month	[44]
Nilotinib 2nd line 400 mg bid	$10,436.00 per month	[44]
Bosutinib 2nd line 500 mg qd	$11,277.36 per month	[44]
Ponatinib 2nd line 45 mg qd	$12,611.04 per month	[44]
Hydroxyurea 2000 mg qd	$655.24 per month	[44]
Tacrolimus 2 mg/day	$313.20 per month	[44]
Mycophenolate 2000 mg/day	$1,887.35 per month	[44]
*Therapy cost*		
Outpatient in CP	$162.52 per month	[40, 45]
Inpatient in CP	$323.09 per month	[40, 45]
Outpatient in AP	$261.98 per month	[40, 45]
Inpatient in AP	$2,173.10 per month	[40, 45]
Outpatient in BP	$261.98 per month	[40, 45]
Inpatient in BP	$1,890.59 per month	[40, 45]
*Stem-cell transplantation and its complication*		
Acute GvHD	$66,821.50	[46]
Chronic GvHD	$10,082.11	[46]
Follow-up care within the first year after SCT	$556.15 per month	[40, 45], clinical expert opinion
Follow-up care beyond the first year after SCT	$485.61 per month	[40, 45], clinical expert opinion
Transplant from live related donor (occurs just once)	$90,234.54	[47]
Transplant from live unrelated donor (occurs just once)	$131,976.34	[47]
*Adverse events*		
Abdominal pain	$5,176.34 per inpatient stay	[47]
Anemia	$4,919.15 per inpatient stay	[47]
Diarrhea	$5,389.65 per inpatient stay	[47]
Hypertension	$6,845.76 per inpatient stay	[47]
Leukocytopenia	$6,424.44 per inpatient stay	[47]
Neutropenia	$8,400.54 per inpatient stay	[47]
Pancreatitis	$7,656.35 per inpatient stay	[47]
Rash	$3,915.32 per inpatient stay	[47]
Thrombocytopenia	$5,846.95 per inpatient stay	[47]

AP: accelerated phase; bid: twice a day; BP: blast phase; CML: chronic myeloid leukemia; CP: chronic phase; GvHD: graft-versus-host disease; qd: every day; SCT: stem cell transplantation.

**Table 3:** Cost-effectiveness results base-case analysis.

	Costs ($)	Life years	QALYs	ICERs ($/LY)	ICURs ($/QALYs)
Chemo	94,492	4.86	3.47	—	—
Bosutinib → chemo/SCT	676,243	9.06	6.86	Weakly dominated	Weakly dominated
Imatinib → chemo/SCT	749,272	9.61	7.29	137,900	171,700
Nilotinib → chemo/SCT	884,222	10.08	7.65	Weakly dominated	Weakly dominated
Dasatinib → chemo/SCT	912,367	10.02	7.61	Dominated	Dominated
Bosutinib → nilotinib → chemo/SCT	913,682	9.96	7.82	Dominated	Weakly dominated
Bosutinib → ponatinib → chemo/SCT	947,136	9.92	7.80	Dominated	Dominated
Imatinib → nilotinib → chemo/SCT	965,597	10.44	8.14	260,800	253,500
Imatinib → ponatinib → chemo/SCT	995,868	10.40	8.12	Dominated	Dominated
Imatinib → bosutinib → chemo/SCT	1,020,857	10.57	8.22	Weakly dominated	Weakly dominated
Bosutinib → dasatinib → chemo/SCT	1,062,220	10.45	8.14	Dominated	Dominated
Imatinib → dasatinib → chemo/SCT	1,099,065	10.88	8.43	Weakly dominated	Weakly dominated
Nilotinib → ponatinib → chemo/SCT	1,108,291	10.80	8.39	Dominated	Dominated
Dasatinib → nilotinib → chemo/SCT	1,111,549	10.79	8.38	Dominated	Dominated
Nilotinib → bosutinib → chemo/SCT	1,130,750	10.95	8.48	Weakly dominated	Weakly dominated
Dasatinib → ponatinib → chemo/SCT	1,139,314	10.75	8.35	Dominated	Dominated
Dasatinib → bosutinib → chemo/SCT	1,162,092	10.90	8.45	Dominated	Dominated
Nilotinib → dasatinib → chemo/SCT	1,200,921	11.23	8.67	299,800	445,100

Chemo: chemotherapy; ICERs: incremental cost-effectiveness ratios; ICURs: incremental cost-utility ratios; QALY: quality-adjusted life years; SCT: stem-cell transplantation.

**Table 4:** Scenario analysis generic pricing imatinib.

Scenario	A: base-case			B: imatinib 60% of original cost			C: imatinib 40% of original cost		
	Cost (US$)	Effectiveness (QALYs)	ICUR (US$/QALY)	Cost (US$)	Effectiveness (QALYs)	ICUR (US$/QALY)	Cost (US$)	Effectiveness (QALYs)	ICUR (US$/QALY)
Chemo	94,492	3.47		94,492	3.47		94,492	3.47	
Imatinib → chemo/SCT	749,272	7.29	171,700	510,214	7.29	109,000	390,685	7.29	77,600
Imatinib → nilotinib → chemo/SCT	965,597	8.14	253,500	726,539	8.14	253,500	607,010	8.14	253,500
Imatinib → dasatinib → chemo/SCT	1,099,065	8.43	—	860,007	8.43	463,800	740,477	8.43	463,800
Nilotinib → dasatinib → chemo/SCT	1,200,921	8.67	445,100	1,200,921	8.67	1,415,200	1,200,921	8.67	1,911,400

Chemo: chemotherapy; QALY: quality-adjusted life years; SCT: stem-cell transplantation.
