# Supplementary material for: Effectiveness and Cost-Effectiveness of Sequential Treatment of Patients with Chronic Myeloid Leukemia in the United States: A Decision Analysis
Source: Leuk Res Treatment. 2015 Dec 10;2015:982395. doi: 10.1155/2015/982395 (PMC4689963; doi:10.1155/2015/982395)
Supplement: Supplementary file 1 — The supplementary appendix gives an additional overview on modeling results. Table S1 shows the results of the effectiveness results of the base-case analysis without discounting and Table S2 provides the results of a scenario analysis varying the second-line TKI effectiveness. [file 982395.f1.docx]

**Effectiveness and Cost-Effectiveness of Sequential Treatment of Patients with Chronic Myeloid Leukemia in the United States - A Decision Analysis**

Ursula Rochau^1,2^; Martina Kluibenschaedl^1^; David Stenehjem^3,4^; Kuo Kuan-Ling^3^; Jerald Radich^5^; Gary Oderda^3^; Diana Brixner^1,2,3,6^; Uwe Siebert^1,2,7,8^

^1^Institute of Public Health, Medical Decision Making and Health Technology Assessment, Department of Public Health and Health Technology Assessment, UMIT - University for Health Sciences, Medical Informatics and Technology, Eduard Wallnoefer Center 1, A-6060 Hall in Tirol, Austria

^2^Area 4 Health Technology Assessment and Bioinformatics, ONCOTYROL - Center for Personalized Cancer Medicine, Karl-Kapferer-Straße 5, 6020 Innsbruck, Austria

^3^Department of Pharmacotherapy, University of Utah, 30 South 2000, Salt Lake City, UT 84112, USA

^4^Huntsman Cancer Institute, University of Utah Hospitals & Clinics, 2000 Circle of Hope, Salt Lake City, UT 84112, USA

^5^Clinical Research Division, Fred Hutchinson Cancer Research Center, 1100 Fairview Ave N, Seattle, WA 98104, USA

^6^Program in Personalized Health, University of Utah, 15 North 2030 East, Room 2110, Salt Lake City, Utah 84112, USA

^7^Center for Health Decision Science, Department of Health Policy and Management, Harvard T.H. Chan School of Public Health, 718 Huntington Ave., Boston, MA 02215, USA

^8^Institute for Technology Assessment, Department of Radiology, Massachusetts General Hospital, Harvard Medical School, 101 Merrimac Street, Boston, MA 02114, USA

Corresponding Author:

Prof. Dr. Uwe Siebert, MPH, MSc

Area 4 Health Technology Assessment and Bioinformatics,

ONCOTYROL - Centre for Personalized Cancer Medicine

Innrain 66a

A – 6020 Innsbruck

Austria

T: +43(0)50-8648-3930, F: +43(0)50-8648-673930

uwe.siebert@oncotyrol.at

# Table S1: Effectiveness results base-case analysis

|  | **0% discounting** | | | | **3% discounting** | | | |
| --- | --- | --- | --- | --- | --- | --- | --- | --- |
|  | **Life years** | **Delta LYs** | **QALYs** | **Delta QALYs** | **Life years** | **Delta LYs** | **QALYs** | **Delta QALYs** |
| Chemo | 5.43 |  | 3.86 |  | 4.86 |  | 3.47 |  |
| Bosutinib -> chemo/SCT | 11.28 | 5.85 | 8.54 | 4.68 | 9.06 | 4.20 | 6.86 | 3.39 |
| Imatinib -> chemo/SCT | 12.09 | 0.81 | 9.14 | 0.60 | 9.61 | 0.55 | 7.29 | 0.43 |
| Bosutinib -> ponatinib -> chemo/SCT | 12.42 | 0.33 | 9.61 | 0.47 | 9.92 | 0.31 | 7.61 | 0.32 |
| Bosutinib -> nilotinib -> chemo/SCT | 12.48 | 0.06 | 9.68 | 0.07 | 9.96 | 0.04 | 7.65 | 0.04 |
| Dasatinib -> chemo/SCT | 12.70 | 0.22 | 9.71 | 0.03 | 10.02 | 0.06 | 7.80 | 0.15 |
| Nilotinib -> chemo/SCT | 12.79 | 0.09 | 9.75 | 0.04 | 10.08 | 0.06 | 7.82 | 0.02 |
| Imatinib -> ponatinib -> chemo/SCT | 13.16 | 0.37 | 10.21 | 0.46 | 10.40 | 0.32 | 8.12 | 0.30 |
| Bosutinib -> dasatinib -> chemo/SCT | 13.21 | 0.05 | 10.23 | 0.02 | 10.44 | 0.04 | 8.14 | 0.02 |
| Imatinib -> nilotinib -> chemo/SCT | 13.22 | 0.01 | 10.25 | 0.02 | 10.45 | 0.01 | 8.14 | 0.00 |
| Imatinib -> bosutinib -> chemo/SCT | 13.40 | 0.18 | 10.37 | 0.12 | 10.57 | 0.12 | 8.22 | 0.08 |
| Dasatinib -> ponatinib -> chemo/SCT | 13.71 | 0.31 | 10.60 | 0.23 | 10.75 | 0.18 | 8.35 | 0.13 |
| Dasatinib -> nilotinib -> chemo/SCT | 13.76 | 0.05 | 10.63 | 0.03 | 10.79 | 0.04 | 8.38 | 0.03 |
| Nilotinib -> ponatinib -> chemo/SCT | 13.78 | 0.02 | 10.65 | 0.02 | 10.80 | 0.01 | 8.39 | 0.01 |
| Imatinib -> dasatinib -> chemo/SCT | 13.88 | 0.10 | 10.70 | 0.05 | 10.88 | 0.08 | 8.43 | 0.04 |
| Dasatinib -> bosutinib -> chemo/SCT | 13.93 | 0.05 | 10.75 | 0.05 | 10.90 | 0.02 | 8.45 | 0.02 |
| Nilotinib -> bosutinib -> chemo/SCT | 14.00 | 0.07 | 10.80 | 0.05 | 10.95 | 0.05 | 8.48 | 0.03 |
| Nilotinib -> dasatinib -> chemo/SCT | 14.44 | 0.44 | 11.10 | 0.30 | 11.23 | 0.28 | 8.67 | 0.19 |

Legend: Chemo, chemotherapy; LYs, life years, QALYs, quality-adjusted life years; SCT, stem cell transplantation;

# Table S2: Scenario analysis

| **Scenario** | **A: Base-case** | | | **2nd line effectiveness dasatinib** | | | **2nd line effectiveness ponatinib** | | |
| --- | --- | --- | --- | --- | --- | --- | --- | --- | --- |
|  | **Cost (US$)** | **Effectiveness (QALY)** | **ICUR (US$/ QALY)** | **Cost (US$)** | **Effectiveness (QALY)** | **ICUR (US$/ QALY)** | **Cost (US$)** | **Effectiveness (QALY)** | **ICUR (US$/QALY)** |
| Chemo | 94,492 | 3.47 |  | 94,492 | 3.47 |  | 94,492 | 3.47 |  |
| Imatinib🡪 chemo/SCT | 749,272 | 7.29 | 171,700 | 749,272 | 7.29 | 171,700 | 749,272 | 7.29 | 171,700 |
| Imatinib🡪 nilotinib 🡪 chemo/SCT | 965,597 | 8.14 | 253,500 | 1,075,028 | 8.43 | 285,500 | 955,830 | 8.12 | 249,400 |
| Nilotinib🡪 dasatinib🡪 chemo/SCT | 1,200,921 | 8.67 | 445,100 | 1,200,921 | 8.67 | 522,600 | 1,085,828 | 8.39 | 481,100 |

Legend: Chemo, chemotherapy; QALY, quality-adjusted life years; SCT, stem cell transplantation;
